# Will Cannabigerol Trigger Neuroregeneration after a Spinal Cord Injury? An In Vitro Answer from NSC-34 Scratch-Injured Cells Transcriptome

**DOI:** 10.3390/ph15020117

**Published:** 2022-01-19

**Authors:** Andrea Valeri, Luigi Chiricosta, Agnese Gugliandolo, Federica Pollastro, Emanuela Mazzon

**Affiliations:** 1 IRCCS Centro Neurolesi “Bonino-Pulejo”, Via Provinciale Palermo, Contrada Casazza, 98124 Messina, Italy; andrea.valeri@irccsme.it (A.V.); luigi.chiricosta@irccsme.it (L.C.); agnese.gugliandolo@irccsme.it (A.G.); 2 Department of Pharmaceutical Sciences, University of Eastern Piedmont, Largo Donegani 2, 28100 Novara, Italy; federica.pollastro@uniupo.it

**Keywords:** CBG, neuronal injury, natural products, NSC-34, neuroprotective agents, axonal regeneration

## Abstract

Spinal cord injury affects the lives of millions of people around the world, often causing disability and, in unfortunate circumstances, death. Rehabilitation can partly improve outcomes and only a small percentage of patients, typically the least injured, can hope to return to normal living conditions. *Cannabis sativa* is gaining more and more interest in recent years, even though its beneficial properties have been known for thousands of years. Cannabigerol (CBG), extracted from *C. sativa*, is defined as the “mother of all cannabinoids” and its properties range from anti-inflammatory to antioxidant and neuroprotection. Using NSC-34 cells to model spinal cord injury in vitro, our work evaluated the properties of CBG treatments in motor neuron regeneration. While pre-treatment can modulate oxidative stress and increase antioxidant enzyme genes, such as *Tnx1*, decreasing *Nos1* post-treatment seems to induce regeneration genes by triggering different pathways, such as *Gap43* via p53 acetylation by *Ep300* and *Ddit3* and *Xbp1* via *Bdnf* signaling, along with cytoskeletal remodeling signaling genes *Nrp1* and *Map1b*. Our results indicate CBG as a phytocompound worth further investigation in the field of neuronal regeneration.

## 1. Introduction

Spinal cord injury (SCI), along with traumatic brain injury (TBI), are two of the largest causes of death and disability worldwide. It is estimated that, every year, 69 million people suffer from TBI and between 250,000 and 500,000 will have to deal with SCI consequences, a burden that will probably be on them for the rest of their life [1,2]. This difference in the incidence of both injuries is probably due to the high mortality of SCI, or the shorter life expectancy of the patient following SCI [3].

SCI can be divided into complete and incomplete, depending on if, after injury, any motor or sensor functionality is spared. If the injury is complete, unfortunately there will not be any sign of neurologic response under the level of injury and this usually correlates with a prognosis worse than the incomplete injury. Laminectomy or removal of bone fragments may enhance, at least in part, the result of incomplete SCI [4]. According to the American Association of Neurological Surgeons (AANS) and the Congress of Neurological Surgeons’ joint section guideline series, the first intervention is assuring the survival of the patient. Apart from the surgery, the treatment involved steroids to upregulate anti-inflammatory cytokines and fight oxidative stress for neuroprotection purposes and the management of blood pressure, because it has been shown that an increase in perfusion in risky tissue is a good neuroprotective strategy [5].

Motor neurons are specialized types of neurons which possess a large variety of functions. In particular, the ones that are located in the ventral horn of the spinal cord, named Spinal Cord Motor Neurons, control the muscles in the periphery of the body and they are the final effectors of the signals coming from the Central Nervous System [6]. These irreplaceable cells are involved in the muscle contraction through a special type of synapse, the Neuromuscular Junction. Due to their characteristics and aim, an injury affecting spinal cord motor neurons results in the impairment of muscle contraction, often leading to partial or complete paralysis [7]. The molecular mechanism of neuronal death in SCI shares some common points with TBI, like the disruption of neurotransmission and, in particular, the over excitation of neurons increases the production of free radicals due to the continuous waves of calcium. The excessive quantity of free radicals is toxic for the injured cells and also the healthy neurons, which might be spared from the physical damage, can suffer this unfavorable environment and die, worsening the outcome of the injury [8].

*Cannabis sativa* is known from ancient times for its beneficial properties and the recent decriminalization of its use has increased the number of people who gain interest in it. The scientific community, however, studied the characteristics of its derivative compounds far before public attention started to argue if there could be something more than Δ^9^-tetrahydrocannabinol (THC) in *C. sativa*: the first phytocannabinoid to be isolated was cannabinol (CBN), whose structure was defined in the 1930s and its synthesis is dated in 1940 [9]. There are many different phytocannabinoids in *C. sativa* and all of them have cannabigerolic acid (CBGA) as a precursor. By heat, CBGA is decarboxylated and becomes CBG. It can bind to cannabinoid receptor 1 (CB1) and cannabinoid receptor 2 (CB2) as a weak agonist and partial agonist, respectively; it is not known whether it can bind to G protein-coupled receptor 55 (GRP55) and, in that case, which type of action it can exert. Regarding transient receptor potential cation channel subfamily V member 1 (TRPV1), another receptor for cannabinoids, CBG appears to have agonist action [10]. Interestingly, some beneficial effects of CBG seem to be mediated by another receptor: peroxisome proliferator-activated receptor gamma (PPARγ). As for VCE-003.2, a quinone derivative of CBG, it can reduce anti-inflammatory cytokines but it can also play a beneficial role in motor neuron preservation in diseases like Huntington’s Disease or Parkinson’s Diseases [10,11].

Previous evidence highlights the effect of cannabinoids in neuroprotection [12,13] and several tests connect both natural and synthetic *C. sativa* compounds with neurogenesis, like VCE-003.2 [14,15], opening new opportunities to explore other beneficial properties of this plant. Our team previously demonstrated the role of CBG in influencing the synaptic pathways and as an anti-oxidant and anti-inflammatory agent [16,17], but its role in the regeneration of neurons is not well understood.

Using the motor-neuron cell model NSC-34, we simulated an acute trauma using scratch injury and test if CBG, administered as pre-treatment or post-treatment, could improve the outcome of damaged neurons and/or can have a role in the axonal regrowth.

## 2. Results

### 2.1. Differentially Expressed Genes Enrichment Analysis and Pathways Inspection

**Figure 1:**
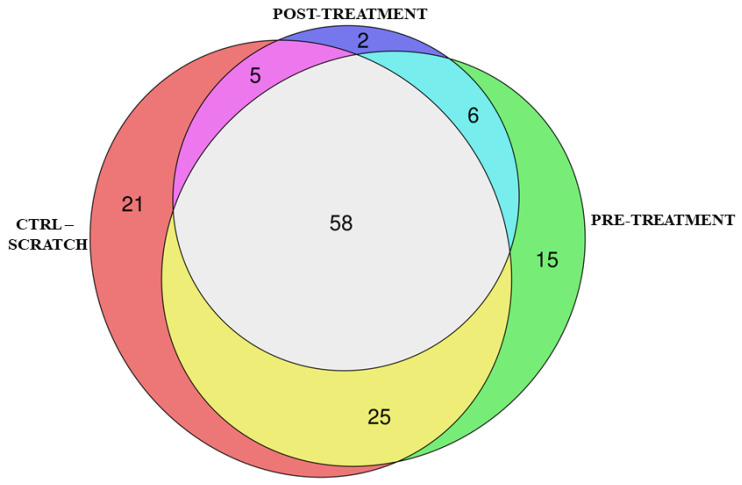
Euler diagram of the overrepresented KEGG pathways between the different comparisons. We observed that 58 pathways are commonly enriched in CTRL against scratch, pre-treatment, and post-treatment. CTRL against Scratch comparison individually enriches 21 pathways. Moreover, six pathways are commonly enriched before and after the treatment with CBG. Additionally, 15 pathways are exclusively enriched with pre-treatment and 2 pathways are specific of the post-treatment.

Differences in Differentially Expressed Genes (DEGs) distribution were obtained in the comparisons between CTRL against Scratch, Scratch against CBG-treated cells before scratch (Pre-treatment), or Scratch against CBG-treated cells after scratch (post-treatment). In detail, the comparison CTRL shows against Scratch 3938 DEGs, among which 2093 are upregulated and 1845 are downregulated. On the other hand, Pre-treatment shows the upregulation of 2021 DEGs and the downregulation of 2009, for a total of 4210 DEGs. Conversely, post-treatment has 3364 DEGs, where 1698 are upregulated and 1666 are downregulated. Then, we enriched the DEGs of each comparison with the KEGG pathways using the library cluster Profiler. Among them, we focus our attention on the 58 pathways enriched in all three comparisons that are highlighted in Figure 1.

The aim of the work was to investigate the effect of CBG on neuroregeneration after a traumatic injury on motor neurons. Among the 58 pathways enriched in the 3 comparisons, we choose “Pathways of neurodegeneration—multiple diseases” (mmu05022) and “Amyotrophic lateral sclerosis” (mmu05014) because they are involved in motor neuron degeneration, highlighting the genes that might be deregulated when a motor neuron is damaged or apoptosis is ongoing. “FoxO signaling pathway” (mmu04068) was inspected because of its involvement in oxidative stress management and apoptosis/survival. Moreover, since we sought for neuroregeneration, we inspected the genes involved in “Regulation of actin cytoskeleton” (mmu04810) and “Axon guidance” (mmu04360).

Each set of genes was evaluated between the different comparisons to understand if CBG could ameliorate the cell response. Since we first damaged the cells with scratch injury, signals of stress response from the cells were expected, so genes involved in apoptosis/survival and stress, oxidative stress, and mitochondria were inspected. The genes that influence apoptosis and survival, along with their fold change, are resumed in Table 1. Fold change of genes involved in stress response, oxidative stress, and mitochondria are resumed in Table 2. Table 3, Table 4, Table 5, Table 6 and Table 7 report the genes involved in the regulation of the different mitochondrial complexes.

The oxidative stress and the survivability of the cells are influenced by the FoxO pathway, which genes we investigated, along with the downstream genes resulting from FoxO activation. Genes involved in this pathway are resumed in Table 8.

We investigated if the cell could regenerate itself after the injury and after the treatment with CBG. We also investigated the genes involved in the remodeling of the cytoskeleton. Genes involved in the neuroregeneration, axonal guidance, and cytoskeleton remodeling are resumed in Table 9.

We also investigated the commonly known receptor with which CBG could interact, in order to understand through which of them CBG could exert its effect on the cell. The genes of the known receptor of CBG and their fold change are resumed in Table 10.

### 2.2. Western Blot

**Figure 2:**
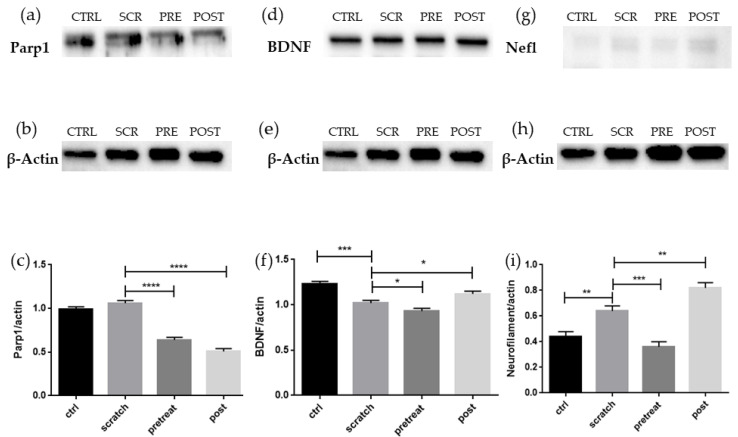
(**a**) Evidence of significant reduction of Parp1 after treatment with CBG, suggesting decreased apoptotic signal. (**b**) β-Actin signal used for normalization. (**c**) Densitometric analysis of Parp1, **** *p* < 0.0001. (**d**) Evidence of significant reduction of BDNF level in scratched cells and increase level in post-treated cells, indicating a positive regenerative and survival signal in post-treated cells. (**e**) β-Actin signal used for normalization. (**f**) Densitometric analysis of BDNF, * *p* < 0.005, *** *p* < 0.0005. (**g**) Evidence of significant increase of Nefl protein in scratched cells and in post-treated cells, while pre-treated cells decrease their Nefl level, suggesting an ongoing axonal regeneration in post-treated cells. (**h**) β-Actin signal used for normalization. (**i**) Densitometric analysis of Nefl, ** *p* < 0.001, *** *p* < 0.0005. CTRL: control; SCR: scratched cells; PRE: pre-treated cells; POST: post-treated cells.

In order to evaluate if the deregulation of the genes analyzed corresponds to a variation also in protein levels, Western Blot was performed on Parp-1, Neurofilament 70 kDa, and BDNF. Results are reported in Figure 2.

Western Blot and statistical analyses showed significant differences in protein level of Parp1 between scratched and treated cells, indicating a reduction in apoptosis signal. The level of BDNF is significantly reduced after scratch and in the pre-treated cells, while post-treated cells showed an increased amount of BDNF protein levels, suggesting that CBG is effective on BDNF only when administered as post-treatment. Light neurofilament protein levels increase after the scratch and decrease in the pre-treated cells, confirming the transcriptomic data, while post-treated cells showed a significantly increased amount of light neurofilament protein levels.

## 3. Discussion

NSC-34 is a cell line widely used as a motor neuron model. It is suitable for the testing of different disease-like conditions, like neurotoxicity [18] as well as neurodegeneration [19]. In this work, using the scratch-injury technique [20,21], we want to mimic the effect of an injury on differentiated NSC-34 cells to answer if the treatment of CBG influences the outcome of traumatic damage and neuronal regeneration. We exposed the cells at CBG at pre-treatment and at post-treatment, after the cells already suffered traumatic injury. The following transcriptomic analysis was performed to elucidate the effects of CBG treatments on the differential expression of motor neuron genes after scratch injury.

As previously reported, oxidative stress and calcium waves are responsible for creating an unfavorable environment for the neurons, which were spared from physical injury, leading them to death. For these reasons, an evaluation on the stress status of the cells and on the main hallmarks of apoptosis was done.

The process of apoptosis is attenuated, but not yet abolished in the pre-treatment group. *Bax* gene expression does not seem to be different compared to scratched cells, where it was upregulated, but after the pre-treatment the pro-apoptotic gene *Parp1* was downregulated [22]. Parp1 protein level does not appear to be significantly different between the control and scratched cells, probably because cell harvest occurred 48 h after the injury was made. However, there is an increasing trend between control and scratched cells. As in the transcriptomic data, Parp1 protein is significantly reduced in pre-treated cells, but it is even more decreased in post-treated cells. Previous studies suggest that inhibition of Parp1 promotes axonal regrowth, but the translation of this model from invertebrate to vertebrates fails to demonstrate the connection between *Parp1* and axonal regrowth, indicating that more in-depth analyses are required [23].

*Pidd1*, encoding for p53-induced death domain protein 1 [24], is downregulated as well and, along with the upregulation of pro-survival genes, like *Birc2* [25], indicates a possible role of CBG pre-treatment in limit of apoptosis signaling. No upregulation of Caspases genes has taken place. Also, comparing the scratched cells with the ones which received CBG as post-treatment, the *Bax* gene does not vary its expression, but *Pidd1* expression is strongly downregulated, indicating that the increase of *Trp53*, encoding for the protein p53, may not be direct to apoptosis. The increased expression of the anti-apoptotic gene *Birc2* level seems to support this hypothesis, along with no increased expression of apoptotic Caspases. The level of *Ddit3* gene, encoding for CHOP, is increased in the post-treatment cells: CHOP increase is usually correlated with ER stress and apoptosis, along with an increased level of *Jun* that, in this comparison, is downregulated [26]; an experiment on primary neurons connects an increase of BDNF level with the subsequent increase of CHOP, indicating an expanded selection of functions for CHOP over apoptosis. The strong increased level of *Xbp1*, indicated as the target of BDNF action via IRE-1α for neurite outgrowth, supports the role of CHOP in BDNF-mediated regeneration, while the downregulation of *Atf4*, part of the apoptotic signal cascade mediated by CHOP, point toward a block of this pathway [27].

There is a link between mitochondrial dysfunction and the onset of ALS, where motor neurons undergo degeneration; since it seems that mitochondrial calcium overload and oxidative stress, among other conditions, can favor the permeability of the mitochondrial membrane, thus leading to cell death [28]. Genes implicated in the calcium flux and oxidative stress were investigated. The expression of *Cycs* does not differ between scratched cells, where it was found upregulated, and pre-treated ones. *Cycs* is a key regulator of mitochondria activity and is suggested to represent an early apoptotic marker [29]. There seemed to be a tentativeness of the cells to balance the expression of the different mitochondria complexes; Cx II is downregulated while Cx IV is upregulated, showing an opposite trend than the one after scratch injury. The expression of the voltage channel coded from the gene *Vdac3* remains upregulated. *Vdac3* seems to have poor control over the redox state of the cells, in contrast its functions seem to be controlled by the redox state [30]. *Mcu* expression is reduced, so that the flow of calcium entering mitochondria could be decreased compared to the scratched cells, where *Mcu* was upregulated. *Gpx4* is downregulated after the scratch injury but, even if *Gpx4* is an antioxidant enzyme and its downregulation might mean a significant increase in oxidative stress [31], the expression of *Nos1* is reduced. *Nos1* encodes for neuronal nitric oxide synthase, so decreasing its level of expression can indicate a cell response by attenuating continuous oxidative stress [32]. Observing the results of post-treated cells’ transcriptome analyses, *Cycs* expression is downregulated, so it might indicate that the level of cytochrome C in the mitochondria is on the way to return to the level before the scratch. To support this evidence, also the expression of almost all mitochondrial complex Cxs is downregulated, suggesting a slight return to normality after the upregulation following scratch injury. Neither *Mcu* nor *Vdac3* expression levels are different from scratched injured cells, and so it is for oxidative stress genes *Gpx4* and *Nos1.*

A pathway involved in SCI is the FoxO one. In the pre-treated cells there is a slight increase of *Foxo3* and *Prkab1*, a subunit of AMPK, which plays its direct role in the FoxO signaling pathway. For this reason, the FoxO pathway involving AMPK action was inspected. Normally AMPK pathway will lead to cell death, caused by the activation of the pro-apoptotic gene *Bcl2l11*, encoding for Bim [33]. However, in our experimental set, Bim is not overexpressed and AP-1, encoded by *Jun* and necessary for apoptosis, is downregulated; therefore, other pathways might be followed [34]. It has been shown that an axis connecting AMPK activation with FoxO3 and their managing of intracellular oxidative stress by enhancing Thioredoxin, a potent antioxidant of the cell [35]; indeed the expression of *Txn1*, encoding for the cytoplasmic Thioredoxin, is strongly upregulated in our experimental set. This is also supported by the evidence that the signal AMPK-FoxO3 is neuroprotective [36]. It was also suggested that FoxO3 can play a role in the management of oxidative stress by inducing autophagy as a mechanism of cell survival [37], so the genes involved in this pathway were also inspected. *Mapk8* is upregulated and seems to be the responsible for the initiation of the FoxO3 signaling towards autophagy; *Bnip3* was shown to protect neuronal cells during oxidative stress by inducing the digestion of unwanted material, which accumulate in the cells [38], and it was found upregulated. Other players in the autophagy process were found upregulated, such as *Atg3*, *Atg4*, *Atg7* and *Atg12*, [39] as well as *Becn1*. The autophagy properties of *Becn1*, encoding for the Beclin-1, can be inhibited by *Bcl2*, downregulated in this comparison, while *Uvrag* is upregulated and its known to interact with Beclin-1 to form the autophagosome [40]. Regarding the post-treated cells, *Foxo3* remains upregulated and so does *Prkab1*, while *Jun* is downregulated, suggesting that the apoptotic pathway signaling in which *Foxo3* is involved is probably not followed. *Atm* is a downstream gene of *Foxo3* [41] and it is known to participate both in apoptosis and in DNA repair. Since apoptosis does not seem to be a followed pathway, it is reasonable to indicate DNA repair as the purpose of *Atm* upregulation. These results point to the capacity of CBG to manage the oxidative stress status of the cells by improving the activity of antioxidant enzymes, reducing the apoptosis signals, regulating the intracellular calcium levels and the over-excitation of neurons. The use of CBG in targeted antioxidant therapies may enhance the survivability of motor neurons after a traumatic injury.

From the axonal regeneration point of view, the markers of neuroregeneration were inspected. Arp2/3 complex acts with N-WASP, encoded by the *Wasl* gene, and they were shown to be active in Golgi polarization in cells close to the border of a wound of a culture of fibroblasts [42]. Even though the Arp2/3 complex is important during neurodevelopment [43], it has been shown that its inhibition, instead of overexpression, leads to axon elongation in primary neurons, suggesting that this complex is a growth cone negative regulator [44,45]. *Arpc2*, *Actr2*, and *Actr3* are the genes of complex Arp2/3 found upregulated in scratched cells and in pre-treatment groups, as well as *Wasl*. *Gap43* correlates with regeneration and it is often used as a classical marker for the growth cone and axon regeneration [46], as well as *Prph*, encoded for peripherin, whose expression varies during axonal regeneration [47]. *Gap43* and *Prph* were downregulated in pre-treated cells. *Nrp1* codes for neuropilin-1, which was found downregulated in scratched cells, and its collateral pruning function seemed important in facilitating the recovery of motor neurons after a traumatic injury [48]. *Map1b* knockdown does not affect the amount of regeneration per se, however it plays a role in the cytoskeleton organization in the new-formed branch. Both *Mapb1* and *Nrp1* are upregulated in the pre-treatment. Taken together, these results suggest that the cell might be managing its internal oxidative stress and could be on the path to balance it, because it seems to be preparing the signals to initiate the regeneration. On the other hand, other evidence suggests that CBG treatment after scratch injury is efficient for trigger regeneration signals. *Gap43* was found to be upregulated, along with *Prph*. *Nrp1* and *Map1b* upregulation points toward the idea that cytoskeleton remodeling is taking place. *Atf3*, upregulated in this comparison, is known to be induced in dorsal root ganglion neurons after injury, but it also promotes the axonal regeneration [49]. It was proposed as an interaction between *Atf3* and *Sox11*, upregulated in post-treated cells, in the regeneration of peripheral injured nerves [50]. Since cytoskeleton remodeling seems influenced by the administration of CBG, genes involved in the architecture of the cytoskeleton were inspected. *Nefl* was upregulated after scratch, while the pre-treatment seems to reduce its expression. The stoichiometry between the different neurofilament chains (Neurofilament Light Chain, encoded by *Nefl* gene; Neurofilament Medium Chain, encoded by *Nefm* gene; Neurofilament Heavy Chain, encoded by *Nefh* gene) is strictly regulated, at least in normal healthy neurons [51]. The downregulation of *Nefl* in pre-treated cells could be interpreted as an attempt of the cell to balance the broken stoichiometry. On the other hand, the expression of *Nefm* is downregulated as well, again leading to the imbalance of the amount of neurofilament chains. *Tardbp*, which encodes for the protein TDP-43, remained upregulated, as was already seen after the scratch injury. It has been suggested its capacity to bind to human NFL and form cytosolic aggregates and, in such a way, disturb the balance in neurofilaments stoichiometry [52,53]. The cytoplasmic accumulation of TDP-43, which gains toxic function, is known to deregulate the mRNA translation, so probably the decrease of *Nefm* expression is correlated with the increase of TDP-43 [54,55]. Similar to the previous comparison, *Nefl* is downregulated also in the post-treatment, but *Nefh* was upregulated. Even though the heavy chain of neurofilaments could undergo the same fate as *Nefl* in TDP-43 granule accumulation, it is worth noting that no upregulation of *Tardbp* gene is taking place. Moreover, evidence suggests a possible role of neurofilament-heavy chain increase mediated by *Bdnf* during regeneration, and this could be mediated also by HuD, encoded from the gene *Elavl4*; in our experiment, *Nefh* is upregulated and so is *Bdnf*, while *Elavl4* is downregulated. The explanation of this apparently weird scenario is given by the timeline of upregulation observed in an experiment of HIV-neuropathy. After ddC injection, the first increase observed was of the BDNF protein level, one day after the injection, while HuD and NF-H followed after three days [56]. It is reasonable to think that NF-H, involved in cytoskeleton organization, is already upregulated because of a regenerative process already started. This hypothesis is reinforced by Western Blot analyses; the protein level of *Nefl* increases after scratch and decreases in the pretreated cells, as found in the transcriptomic analyses, but then it increases again in the post-treated cells, apparently in contrast with the transcriptomic data. It is important to note that, during axonal regeneration, the first upregulated genes are intein and peripherin, along with *Nefl*, followed by *Nefm* and *Nefh* is the last to be overexpressed [57]. This correlates very well with our transcriptomic data. Since *Nefl* is the first neurofilament gene to be expressed, it is reasonable to think that its protein has already been synthesized when *Nefh* started to be expressed, as in the case of post-treated cells. Indeed, the level of BDNF protein is also higher in post-treated cells compared to the scratched ones, connecting *Bdnf* and neurofilaments with the path of axonal regeneration.

Since several signals point toward axonal regeneration, a deeper analysis on the mechanisms, pathways, and genes involved in this important process were done, to give a more complete scenario in the post-treated cells. As mentioned before, *Trp53*, encoding for the protein p53, is upregulated. P53 is involved in a large variety of processes, ranging from apoptosis to DNA repair [58], but also neuroregeneration. *Gap43* is a target gene of p53, which is acetylated by p300 [59], encoding by *Ep300*, upregulated in our experiment; it was also noticed that knocking down p53 has the potential to decrease the regeneration of nerve fibers in mice and its overexpression has the capacity to increase the size of the growth cone [60], which could very well correlate with *Nefh* upregulation. Other genes inducible by p53 and involved in axonal regeneration or guidance are *Slit2*, *Slit3*, *Neo1*, *Unc5b* and *Ephb3*.

*Unc5b* is primarily known for its role in apoptosis, since its interaction with Caspase-3, which is not deregulated in this comparison, mediates cell death [61]. An experiment involving peripheral nerve injury in mice evaluated the role of *Unc5b* in regeneration, since it is the receptor of Netrin-1; the *Unc5b* signal acts as a repulsive signal, so it will repel axon growth cones in order to help it to reach the right target. In the mentioned experiment, UNC5B protein level peaks 14 days after injury and the authors interpret this as the time when the regenerating axon passed over the analyzed nerve fragment [62]. In our experimental comparison, the *Unc5b* level is decreased. We harvest the cells long before 14 days, so it is reasonable to think that there was no need for repulsive signals since no axons had the time to reach the target. *Slit2* and *Slit3* are important components of SLIT/ROBO signaling, necessary for the control of the axon pathfinding. SLITs are the secreted protein and ROBOs are their receptors. An experiment of sciatic nerve crush in mice revealed the downregulation of *Slit2* after injury, but also its upregulation during axonal regeneration, which could be the case of our comparison where *Slit2* is slightly upregulated [63]. The downregulation of the repellent signal from *Slit3* may be interpreted as the *Unc5b* one, so the regeneration has just started and there is no point to repel an axon that is not already grown. Moreover, there is no change in *Robos* genes expression, suggesting that probably no interaction between SLITs and ROBOs is taking place. *Neo1* is a transmembrane receptor, and it is involved in axon guidance and is downregulated in this comparison. RGMA and NET1 promote the formation of a complex known to mediate the collapse of the growth cone [64], so the downregulation of *Neo1* could be interpreted as a positive signal of ongoing axonal regeneration. *Ephb3* is downregulated in post-treated cells as well. This belongs to another complex of signals because its ligand is Ephrin3B, encoded by *Efnb3*, upregulated in our study, and their interaction can have different outcomes. They play a role in suppressing proliferation in neural stem cells and *Ephb3* can also induce cell death [65]. It is important to notice that Ephrin3B presence seems to attenuate the cell death after adult TBI in mice [66]. Taken together, this evidence points to an important role of p53 and *Bdnf* in regenerating damaged motor neurons after CBG exposure, making them possible targets for future therapies. A careful management of these pathways through CBG administration could be useful to increase the regeneration of neurons after traumatic injuries.

**Figure 3:**
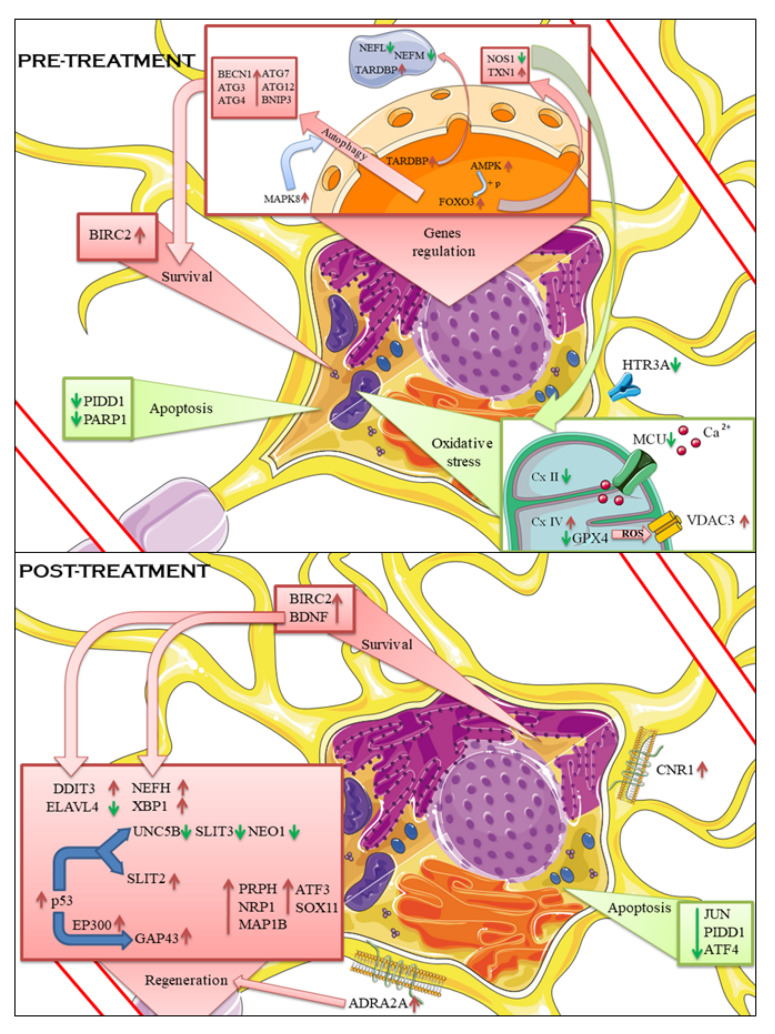
DEGs comparison between the two treatments with the biological process associated with them. Impaired processes and downregulation of genes are reported in green. Enhanced processes and upregulation of genes are reported in red. Pretreating the cells with CBG makes them more resistant to the outcomes of scratch injury. The regulation of oxidative stress seems triggered by FoxO3, which can also promote autophagy to get rid of the toxic aggregates in the cells, enhancing the survival of the cells. Treating the cells with CBG after scratch injury increases survival signaling while decreasing apoptotic ones. The amount of regeneration genes upregulated strongly points to a regenerative process occurring. *Bdnf* and p53 seem to be the main triggers of this process. The figure was drawn using the vector image bank of Servier Medical Art by Servier (smart.servier.com, accessed on 14 December 2021). It is licensed under a Creative Commons Attribution 3.0 Unported License (https://creativecommons.org/licenses/by/3.0/, accessed on 14 December 2021).

The NSC-34 cell line does not express *Cnr2*, which encodes for the Cannabinoid Receptor 2 [67], while it does have *Cnr1*, the gene for the Cannabinoid Receptor 1. CBG can normally exert its action on both with a lower affinity than CBD [68], but the pre-treatment seems not to upregulate *Cnr1* gene expression. The treatment of CBG after the injury appears to increase the expression level of *Cnr1*, while the scratch injury, without any other treatment, appears to decrease its expression compared to control cells. Another receptor with which CBG could interact is PPAR-γ, but neither the pre-treatment nor the treatment after injury upregulate *Pparg* gene expression. CBG proved to be a potent agonist of α-2 adrenoceptor, encoded from the gene *Adra2a*, upregulated in the post-injury treatment, and it is a mild antagonist of the 5HT_1A_ receptor, which is a serotoninergic receptor encoded from the gene *Htr1a* [69]. Even though no difference in this latter gene expression was found, it is worth mentioning that the pre-treatment strongly downregulates the *Htr3a* gene, which encodes for the subunit A of the serotonin type 3 receptor. Even if there is no evidence in the literature connecting adrenoceptors and axonal regeneration in motor neurons, an experiment about optic nerve injury in rats elucidates the capacity of α-2 adrenoceptor agonists to promote axonal growth of retinal ganglion cells [70]. Since, in our experimental set, post-injury CBG treatment seems to upregulate genes involved in the axonal regeneration, it is possible that an α-2 adrenoceptor action could trigger axonal regeneration in motor neurons. Further analyses could better clarify this association. Another experiment aimed to evaluate the mechanism behind the evidence that antagonists of the serotonin 3 receptor (5-HT_3_R) have anti-apoptotic and anti-inflammatory effects. The proposed mechanism involved the inhibition of TNF-α pathway via antagonism of 5-HT_3_R [71] and this might be connected with our evidence that CBG pre-treatment reduced the expression of *Tnfaip1*. This gene encodes for the TNF Alpha Induced Protein 1, identified as a protein which can be induced by TNF-α [72]. The results of our experiment are reported in Figure 3.

## 4. Materials and Methods

### 4.1. NSC-34 Culture, Differentiation, and Treatment

The NSC-34 cell line was thawed from a stock vial obtained from Cedarlane Corporation (Burlington, ON, Canada). The maintenance of the cell line was done following the datasheet from the company. Briefly, the culture medium is composed of DMEM High Glucose, 10% Fetal Bovine Serum, 1% penicillin/streptomycin, and 1% L-Glutamine. All reagents were purchased from Sigma-Aldrich, Merck KGaA (Darmstadt, Germany). The differentiation medium is composed by 1:1 DMEM/F-12 (Ham), 1% Fetal Bovine Serum, 1% L-Glutamine, 0.5% penicillin/streptomycin and retinoic acid at the concentration of 1 μM.

Cells were seeded in 6 well plates and 24 h after seeding, the culture medium was all replaced with differentiation medium, and was changed every 2 days. Five days after differentiation treatment, cells underwent different treatment paradigms.

Cells were divided into different groups:

(1) Control: no treatment or injury
(2) Scratch: using a 1 mL pipette tip, cells were manually scratched. A total of four horizontal and four vertical scratches were made and the space between each scratch was approximately 2 mm
(3) Pre-treatment: before the scratch injury was made, cells were exposed for 24 h to DMEM high glucose + 1% FBS + 1% Glutamine + 0.5% penicillin/streptomycin + 7.5 μM CBG
(4) Post-treatment: after the scratch injury was made, cells were exposed for 24 h to DMEM high glucose + 1% FBS + 1% Glutamine + 0.5% penicillin/streptomycin + 7.5 μM CBG
(5) CBG: cells were not scratched, but the medium was changed to DMEM high glucose + 1% FBS + 1% Glutamine + 0.5% penicillin/streptomycin + 7.5 μM CBG.

The concentration of CBG was based on previous evidence obtained from our laboratory regarding the minimum dose of CBG that can produce any effect on cells [17,73]. Every change of medium in one group corresponded a medium change or replacement in all the other groups, in order to be confident that any observed effect was due to the treatment and not to lack of nutrients. Cells were collected 24 h after post-injury CBG treatment.

### 4.2. CBG Purification

CREA-CIN (Rovigo, Italy) supplies *Cannabis sativa* in compliance with their legal status (authorization SP/106 23/05/2013 of the Ministry of Health, Rome, Italy). According to the protocol which avoids Δ^9^-THC isolation and extraction, CBG was isolated and purified in agreement with the standardized protocol, in order to reach over 99% or purity [17,74].

### 4.3. RNA Isolation from Cells Pellet and cDNA Library Preparation

Maxwell^®^ RSC simplyRNA Cells Kit (Promega, Madison, WI, USA) was used to extract DNA from cell pellets after harvesting, following the manufacturer’s instructions. As previously reported, the library was prepared using TruSeq RNA Exome protocol (Illumina, San Diego, CA, USA) [75].

### 4.4. Call of Differentially Expressed Genes

The raw data obtained used the MiSeq instrument of Illumina were analyzed through fastQC (version 0.11.5, Babraham Institute, Cambridge, UK) and adapters and bases with low scores were removed with Trimmomatic (version 0.38, Usadel Lab, Aachen, Germany) [76]. The mouse reference genome GRCm39 was downloaded from the Gencode website [77,78] and the related index was created for the Spliced Transcripts Alignment to a Reference (STAR) RNA-seq aligner (New York, NY, USA) [79] using the annotation version M27.

The analysis of differentially expressed genes (DEGs) was performed with the Bioconductor package DESeq2 [80] in R (version 3.6.3, R Core Team) after the count call provided by htseq-count (version 0.6.1p1, European Molecular Biology Laboratory (EMBL), Heidelberg, Germany) [81] for each comparison. The Benjamini–Hochberg post hoc correction was computed to discard false positive DEGs using a q-value threshold of 0.05 so no fold change threshold was included. The clusterProfiler library [82] of Bioconductor was used to enrich DEGs by KEGG pathways and Gene Ontology Biological Processes.

### 4.5. Protein Isolation and Western Blot

At the end of the treatment, a single 6-well plate was used for protein extraction. The cells from different treatment paradigms were harvested and protein was extracted following NE-PER Nuclear and Cytoplasmic Extraction reagents protocol (Thermo Scientific, Pierce Biotechnology product n. 78833, Rockford, IL, USA). The cytoplasmic protein concentration was addressed using Bradford Assay (Bio-Rad, Hercules, CA, USA). Proper dilutions were made to reach a total value of 30 µg of protein per sample. Samples were denaturized at 95 °C and SDS-polyacrylamide gel electrophoresis (SDS-PAGE) was performed before the transfer of the proteins on a PVDF membrane (Immobilon–P, Millipore, Burlington, MA, USA). The membranes were blocked in 5% skim milk in TBS for 1 h at room temperature followed by an overnight incubation at 4°C with the following primary antibodies: anti-BDNF (1:1000, Abcam, Cambridge, UK, ab108319), anti-Parp-1 (1:200, Santa Cruz Biotechnology, Dallas, TX, USA, sc-25780), anti-Neurofilament 70 KDa (1:1000, Chemicon International, Hessen, DE, MAB1615) and anti-β-Actin (1:1000, Santa Cruz Biotechnology, Dallas, TX, USA, sc-47778). The membranes were then washed with TBS 1x and incubated with the proper secondary antibodies for 1 h at room temperature: mouse anti-rabbit IgG-HRP (1:2000, Santa Cruz Biotechnology, Dallas, TX, USA, sc-2357) and chicken anti-mouse IgG (1:2000, ThermoFisher Scientific, Waltham, MA, USA, SA1-72021). The bands were then acquired using the ChemiDoc™ MP System (Bio-Rad) after being exposed to an enhanced chemiluminescence system (Luminata Western HRP Substrates, Millipore, Burlington, MA, USA).

## 5. Conclusions

Treating the cells with CBG before the scratch injury mitigates apoptosis signaling and proved to be effective in the management of oxidative stress. Treating the cells with CBG after the scratch injury demonstrates to be efficient in inducing neuroregeneration genes while upregulating survival signaling, impairing apoptosis process.

CBG proved in such a way to be a promising candidate in future studies involving neuronal regeneration. Future directions point toward the optimization of the protocol, where different doses of CBG could lead to different results and adjust the length of the treatment. Testing the combination with regenerative therapies or drugs may lead to surprising results.

## Figures and Tables

**Table 1:** DEGs involved in apoptosis/survival.

Gene	Process	CTRL—Scratch	Pre-Treatment	Post-Treatment
*Bax*	Apoptosis	0.42	-	-
*Parp1*		0.13	−0.30	-
*Pidd1*		-	−1.96	−2.44
*Jun*		0.54	−0.46	−0.40
*Atf4*		−0.07	-	−0.10
*Birc2*	Survival	-	0.62	0.36
*Bdnf*		−0.99	-	0.88

The fold change is computed as log_2_(Scratch/CTRL) for CTRL against Scratch comparison, as log_2_(Pre-treatment/Scratch) for Pre-treatment comparison, as log_2_(Post-treatment/Scratch) for Post-treatment comparison. The values are rounded to the second decimal digit.

**Table 2:** DEGs involved in stress response, oxidative stress, and mitochondria.

Gene	CTRL—Scratch	Pre-Treatment	Post-Treatment
*Cycs*	0.55	-	−0.56
*Vdac3*	0.35	0.23	-
*Gpx4*	−0.28	−0.22	-
*Nos1*	-	−0.58	-
*Txn1*	-	1.62	-
*Mcu*	0.57	−0.41	-
*Tardbp*	0.15	0.28	-

The fold change is computed as log_2_(Scratch/CTRL) for CTRL against Scratch comparison, as log_2_(Pre-treatment/Scratch) for Pre-treatment comparison, as log_2_(Post-treatment/Scratch) for Post-treatment comparison. The values are rounded to the second decimal digit.

**Table 3:** DEGs involved in regulation of Cx I.

Gene	CTRL—Scratch	Pre-Treatment	Post-Treatment
*Ndufs1*	0.12	-	−0.14
*Ndufs2*	-	0.11	0.07
*Ndufs3*	0.24	-	-
*Ndufs4*	0.50	-	−0.18
*Ndufs7*	-	−0.45	-
*Ndufs8*	-	−1.92	−0.89
*Ndufv1*	0.41	−0.31	−0.15
*Ndufv2*	0.19	0.22	-
*Ndufa3*	-	−0.66	-
*Ndufa5*	−1.69	-	-
*Ndufa6*	-	0.30	-
*Ndufa12*	-	-	−0.39
*Ndufab1*	0.25	0.16	-
*Ndufb6*	-	0.77	-
*Ndufb7*	-	−0.61	-
*Ndufb11*	0.30	-	−0.28
*Ndufc1*	-	5.38	-

The fold change is computed as log_2_(Scratch/CTRL) for CTRL against Scratch comparison, as log_2_(Pre-treatment/Scratch) for Pre-treatment comparison, as log_2_(Post-treatment/Scratch) for Post-treatment comparison. The values are rounded to the second decimal digit.

**Table 4:** DEGs involved in regulation of Cx II.

Gene	CTRL—Scratch	Pre-Treatment	Post-Treatment
*Sdha*	0.24	-	−0.14
*Sdhb*	0.22	−0.34	−0.27

The fold change is computed as log_2_(Scratch/CTRL) for CTRL against Scratch comparison, as log_2_(Pre-treatment/Scratch) for Pre-treatment comparison, as log_2_(Post-treatment/Scratch) for Post-treatment comparison. The values are rounded to the second decimal digit.

**Table 5:** DEGs involved in regulation of Cx III.

Gene	CTRL—Scratch	Pre-Treatment	Post-Treatment
*Uqcrfs1*	0.37	-	-
*Cyc1*	0.22	-	-
*Uqcrb*	0.35	0.35	-
*Uqcr10*	3.54	-	−2.93
*Uqcr11*	−0.46	-	-

The fold change is computed as log_2_(Scratch/CTRL) for CTRL against Scratch comparison, as log_2_(Pre-treatment/Scratch) for Pre-treatment comparison, as log_2_(Post-treatment/Scratch) for Post-treatment comparison. The values are rounded to the second decimal digit.

**Table 6:** DEGs involved in regulation of Cx IV.

Gene	CTRL—Scratch	Pre-Treatment	Post-Treatment
*Cox4i2*	-	1.46	-
*Cox5b*	-	-	0.27
*Cox6a1*	−0.46	0.46	-
*Cox6c*	-	−4.89	-
*Cox7c*	−0.41	0.51	-

The fold change is computed as log_2_(Scratch/CTRL) for CTRL against Scratch comparison, as log_2_(Pre-treatment/Scratch) for Pre-treatment comparison, as log_2_(Post-treatment/Scratch) for Post-treatment comparison. The values are rounded to the second decimal digit.

**Table 7:** DEGs involved in regulation of Cx V.

Gene	CTRL—Scratch	Pre-Treatment	Post-Treatment
*Atp5a1*	-	−0.14	-
*Atp5b*	0.07	-	−0.12
*Atp5c1*	0.17	0.21	-
*Atp5pb*	0.34	-	-
*Atp5g1*	0.36	-	−0.08
*Atp5g3*	0.21	0.10	-
*Atp5g2*	-	0.17	-
*Atp5l*	0.14	0.33	-
*Atp5h*	−0.51	-	-
*Atp5k*	−4.52	-	-
*Atp5l*	0.14	0.33	-
*Atp5j*	0.29	-	-
*Atp6v1b2*	-	0.23	-
*Atp6v1c1*	-	0.21	-
*Atp6v1d*	-	0.39	0.24
*Atp6v1e1*	-	0.36	-
*Atp6v1g1*	-	−0.23	0.20
*Atp6v1g2*	-	-	1.13
*Atp6v1h*	-	0.30	-
*Tcirg1*	−0.65	-	-
*Atp6v0d1*	−0.18	-	-
*Atp6v0b*	-	-	0.18
*Atp6v0c*	-	−0.45	-
*Atp6ap1*	-	-	0.25

The fold change is computed as log_2_(Scratch/CTRL) for CTRL against Scratch comparison, as log_2_(Pre-treatment/Scratch) for Pre-treatment comparison, as log_2_(Post-treatment/Scratch) for Post-treatment comparison. The values are rounded to the second decimal digit.

**Table 8:** DEGs involved in FoxO pathway.

Gene	CTRL—Scratch	Pre-Treatment	Post-Treatment
*Foxo3*	-	0.07	0.08
*Prkab1*	-	0.61	0.37
*Mapk8*	0.38	0.19	−0.34
*Bnip3*	0.22	0.11	−0.53
*Atg3*	-	0.23	-
*Atg4a*		0.61	
*Atg7*	−0.93	1.45	0.75
*Atg12*	-	0.85	-
*Becn1*	-	0.10	0.09
*Bcl2*	-	−0.61	-
*Uvrag*	−0.38	0.68	0.59
*Atm*	-	0.43	0.34

The fold change is computed as log_2_(Scratch/CTRL) for CTRL against Scratch comparison, as log_2_(Pre-treatment/Scratch) for Pre-treatment comparison, as log_2_(Post-treatment/Scratch) for Post-treatment comparison. The values are rounded to the second decimal digit.

**Table 9:** DEGs involved in neuroregeneration, axonal guidance, and cytoskeleton remodeling.

Gene	CTRL—Scratch	Pre-Treatment	Post-Treatment
*Arpc2*	0.09	0.08	−0.09
*Actr2*	0.28	0.08	−0.12
*Actr3*	0.25	0.25	−0.18
*Wasl*	0.16	0.29	−0.16
*Gap43*	-	−0.20	0.10
*Prph*	-	−0.30	0.16
*Nrp1*	−0.18	0.50	0.48
*Map1b*	−0.15	0.17	0.19
*Elavl4*	0.14	0.14	−0.18
*Trp53*	-	-	0.36
*Ep300*	−0.05	−0.06	0.15
*Ddit3*	−0.38	-	0.34
*Xbp1*	-	-	1.27
*Atf3*	-	1.05	0.83
*Sox11*	−0.33	−0.43	0.18
*Slit2*	-	-	0.13
*Slit3*	−0.10	−0.26	−0.10
*Neo1*	0.07	-	−0.07
*Unc5b*	−0.30	-	−0.33
*Ephb3*	-	−1.29	−1.06
*Efnb3*	-	-	1.08
*Nefl*	0.36	-1.04	−0.41
*Nefm*	-	-0.35	-
*Nefh*	-	-	0.16

The fold change is computed as log_2_(Scratch/CTRL) for CTRL against Scratch comparison, as log_2_(Pre-treatment/Scratch) for Pre-treatment comparison, as log_2_(Post-treatment/Scratch) for Post-treatment comparison. The values are rounded to the second decimal digit.

**Table 10:** DEGs involved in receptors of CBG.

Gene	CTRL—Scratch	Pre-Treatment	Post-Treatment
*Cnr1*	−0.18	-	0.12
*Trpv1*	−4.03	-	-
*Trpv4*	−0.95	-	-
*Adra2a*	-	-	0.37
*Htr3a*	-	−1.36	-
*Tnfaip1*	-	−0.28	-

The fold change is computed as log_2_(Scratch/CTRL) for CTRL against Scratch comparison, as log_2_(Pre-treatment/Scratch) for Pre-treatment comparison, as log_2_(Post-treatment/Scratch) for Post-treatment comparison. The values are rounded to the second decimal digit.

## Data Availability

The data presented in this study are openly available in the NCBI Sequence Read Archive at BioProject, accession number PRJNA791529.
